# The Week's Work

**Published:** 1911-05-06

**Authors:** 


### The Week's Work.

### GRAVE STATE OF AFFAIRS AT PARKHURST MILITARY HOSPITAL.

The War Office is understood to be making a full inquiry at Parkhurst Military Hospital with reference to the statements arising out of the recent inquest on Sergeant Stokes, R.A.M.C. Sergeant Stokes was dispenser at the hospital, and the jury found that he had committed suicide by poison "whilst in an unsound state of mind produced by ill-health and prolonged hours of duty." The Coroner said that this was the second suicide of a hospital sergeant within nine months. A diary was found on the deceased which ended with these words: "We are all harassed. We can't all be Haldanes. This is the last of an uneventful life spoilt through ill-health and insufficient brain power to combat the duties of ward master, steward and dispenser." Colonel Donegan, commanding the medical staff at Parkhurst, stated that he had repeatedly reported officially on the small numbers of his staff. Two non-commissioned officers who gave evidence admitted working from 7 A.M. till 11 P.M. seven days a week. The Coroner and jury said that "no blame is to be attached to any member of the staff at Parkhurst," but urged the necessity of an official investigation. The widow of the late Sergeant Stokes has since written to the Press saying that Lieut.-Colonel J. D. Francis Donegan has been relieved of his command at Parkhurst, which is to be handed over to a major of his corps. This order from the military authorities seems to us hard to reconcile with the Coroner and jury's statement at the inquest, already quoted, that "no blame is to be attached to any member of the staff." So much money is spent on the Royal Army Medical Corps that, if the apparent evidence of under-staffing so grimly brought forward by the recent inquest prove correct, the commanding officer who has frequently reported it will hardly be encouraged by his present treatment to perform his duties efficiently in whatever other hospital they are required. The result of the inquiry will probably be available for comment next week. The public will certainly expect to know who is responsible.

### THE COMPLETE WARD UNIT.

We publish this week the first of a series of special articles on the complete ward unit for a modern general hospital—a series which we hope will prove of practical value to all interested in hospital construction. This week's plan, which is based on the wards of the Northampton General Hospital, is the combined conception of Dr. Richard Greene and Mr. Frederick Dorman. Both these gentlemen are well known as hospital experts. Dr. Greene, formerly the Superintendent of the Northampton County Asylum, was the joint designer (with Mr. A. Blyth) of the first premium plans for the Derby Borough Asylum and of the first premium plans for the Blackburn and Isle of Wight Asylum, and expert adviser on the plans for the Ilford Hospital. Mr. Dorman, who is an Associate of the Royal Institute of British Architects, has designed several important provincial institutions. The collaboration of two such experts is bound to be productive of highly interesting results. The next article in the series will be by Mr. E. T. Hall, F.R.I.B.A. As we have already stated, we shall be glad to publish any criticism of, or discussion on, the designs with which our readers may favour us.

### MR. JUSTICE JOYCE AND THE DEFINITION OF A GENERAL HOSPITAL.

In our Institutional notes of February 11 we published some remarks on the "New Hospital at Putney," the interest of which is strikingly illustrated by the ruling of Mr. Justice Joyce in the Chancery Division of the High Court last week. In referring to Mr. Chester's bequest, we noted that "Mr. Chester directed that the hospital was to be a general hospital, and that, failing this, the bequest was to revert to Guy's Hospital; but the committee which has this matter in hand will not fail to see that the provisions of the will are carried out, and that the neighbourhood shall benefit by the good intention of the testator." The contention as to whether a hospital is a "General Hospital" if there is no out-patient department was brought before Mr. Justice Joyce on April 25. Although the Judge expressly declined to give the opinion that a hospital was not a "General Hospital" unless it had an out-patient department attached, he went so far as to rule that if the Putney Hospital did not provide an out-patient department, but established a hospital solely for in-patients, the wishes of the testator, Mr. Henry Chester, would not be fulfilled.

### DR. EDWIN GOODALL AND THE CARDIFF NEWSPAPERS.

On the ground that his staff has been charged with self-advertisement owing to the publication of the number of discharges from the Cardiff Mental Hospital, Dr. Goodall is said to have expressed his resentment at the presence of newspaper reporters at the hospital committee meetings. As medical superintendent, he puts down to the eagerness of reporters for copy the accounts of cures at his institution, which, he says, has led to the charges of advertisement being made. The local Press is indignant at his outspoken dislike of its representatives. Whoever made the alleged accusations (and it would be interesting to know), the dispute illustrates the growing feeling among responsible institutional people that the lay papers all the country over tend to regard hospital affairs as "good copy," and shows, if Dr. Goodall's mention of "other Asylum authorities" be correct, that there is a more healthy rivalry than was believed among public institutions. As these do not depend on voluntary subscriptions it is hard to see what the Cardiff Mental Hospital would be supposed to gain by advertisement. The public, as Dr. Goodall must admit, cannot possibly be excluded from knowing what passes at the meetings of public bodies, but in spite of the disclaimer of the *South Wales Daily News*, which we accept, it cannot be denied that the lay Press, at least outside Cardiff, has got the reputation of "Paul Pry and Peeping Tom."

### A NEW FRENCH PRIVATE HOSPITAL.

An interesting experiment is being tried in Paris, by the establishment of a private hospital which is intended to take a place between the ordinary hospitals and the "maisons-de-santé" which charge heavy fees. The new establishment has been started by a civil society with a capital of £32,000, and the building contains 86 beds, of which 40 are for surgical, 26 for medical, and 20 for infectious cases. A radioscopic and radiographic installation has been added for the treatment of both resident and non-resident patients, for the use of which very moderate fees will be charged. The administrative council is composed as follows: Mlle. Louise Chaptal, President; M. Max-Leclerc, secretary; and Mme. la Comtesse de Béarn and M. Edm. Fouret, members. It is hoped that the new institution will prove a boon to that large class of society which, while unwilling to enter the ordinary hospitals because of their being of a more or less charitable origin, are unable to pay the high fees demanded by well-equipped nursing homes. It is however questionable whether the family doctor will welcome the new institution without reserve, since in the reports that we have seen of the new enterprise no mention is made of his being allowed to treat his own patients in the hospital. This may mean yet another grievance to him, in that he will possibly be deprived of patients who in ordinary circumstances would be treated by him in their own homes.

### THE PROBLEM BEFORE THE CHELSEA HOSPITAL FOR WOMEN.

The need for a reconstructed out-patient department and a nurses' home at the Chelsea Hospital for Women was stated in The Hospital of April 15. There the impossibility of lateral extension was also mentioned, but it now remains to point out the exceedingly difficult position in which the Council finds itself—shall it take Mr. Bland Sutton's bold advice and rebuild the whole hospital, or shall it attempt to provide a new out-patient department and a separate nurses' home? The latter course is, relatively speaking, the easy one, for towards its manageable cost is already last year's fortunate accumulation of legacies. It could never be, however, the final solution of the problem, as was felt by the medical staff, which supported Mr. Bland Sutton's proposition. To move and to rebuild no doubt present more than one difficulty, the first of which is the choice of the new site, and the second, which we believe is easily capable of exaggeration, is the cost and construction. A solution cannot be long postponed. While these two plans are under consideration it would be improper for us to discuss the question further, beyond hoping that a unanimous decision may be arrived at, and that the whole matter may be laid before the King's Fund at an early date.

### THE TORQUAY CORPORATION TO APPEAL.

It will surprise no one that the Sanitary Committee of the Torquay Corporation has decided to appeal against the decision of the Torquay County Court, which on April 8 awarded £50 damages against the Corporation for the alleged negligence with which it had treated the son of Mr. W. Gregory, ex-police sergeant, in the borough isolation hospital. In April of last year the boy was taken to the hospital suffering from scarlet fever, in June he was removed to his home, and in December he died from heart disease, which, the plaintiff alleged, was due to the things the boy had been allowed to do at the hospital. The case, it will be seen, is of the greatest importance, as it affects all similar institutions, and the result of the appeal will be awaited with interest.

### THE GERMAN HOSPITAL CONGRESS.

The annual general congress of the German Hospitals Association (Vereinigung d. leitenden Verwaltungs-Beamten v. Krankenanstalten Deutschland) takes place at Dresden from June 25 to 29 next. The Congress is one of the most representative in Europe, and is attended by visitors from all parts of the Continent, so that its proceedings, in view of the animated discussions on papers read, are generally very instructive to hospital workers. The agenda for this year's meeting includes many subjects of paramount importance, among others a debate on the insurance systems and their relations to hospitals. An interesting visiting programme has been arranged. Particulars of the Congress may be had on application to the general secretary, Verwaltungs Direktor Helbig, Liebigstrasse 20, Leipzig. Herr Helbig is the superintendent of the City Infirmary, St. Jacob, at Leipzig, one of the most interesting of the German hospitals, and is one of the editors of our contemporary, *Zeitschrift für Krankenanstalten*.

### THIS WEEK'S DRUG MARKET.

Some improvement in business has taken place since last week, and there has been a fair amount of inquiry for several drugs, and transactions have been on a rather larger scale. Few changes in value, however, have taken place. The high price of opium is well maintained, since it has been definitely ascertained that the extent of the damage to the autumn and winter-sown plants was not exaggerated in the reports from the growing districts; even if the yield from the late-sown poppy seed is above what is expected, the total 1911 crop of opium will be below the average, and although prices might recede to some extent, no material reduction is anticipated. Morphine and codeine are unchanged in price, but in view of the strong position of the opium market a further advance in the value of the alkaloids should not come as a surprise. Cod-liver oil still shows a tendency to decline in value; the yield of oil from cod caught up to the present in the Norwegian fishing grounds is about twenty per cent. below the yield up to the same date last season. Ergot of rye continues very dear. A further small rise in the price of santonin is not altogether unexpected. Buchu leaves are again rather dearer. There has been a slight advance in the price of cascara sagrada, and the tendency still appears to be in an upward direction. Makers of benzoic acid have advanced their price. Importers of quicksilver have made no alteration in price, but another reduction is by no means improbable.

